# Unilateral segmental Darier's disease associated with neuropsychiatric disorders

**DOI:** 10.1002/ccr3.2243

**Published:** 2019-06-03

**Authors:** Luke Horton, Darius Mehregan

**Affiliations:** ^1^ Medical Research Assistant Department of Dermatology and Department of Biomedical Engineering Wayne State University School of Medicine Detroit Michigan; ^2^ Department of Dermatology Wayne State University School of Medicine Detroit Michigan

**Keywords:** Darier's disease, Grover's disease, Hailey‐Hailey

## Abstract

Unilateral segmental Darier's disease (DD) is a rare variant of DD that presents with erythematic lesions in a unilateral distribution without other associated features of DD. Although diagnosis is challenging, unilateral segmental DD should be considered for an acantholytic dermatosis in a unilateral distribution and a history of neuropsychiatric disorders.

## 1 INTRODUCTION

Darier's disease (DD) is a rare autosomal dominant genodermatosis characterized by multiple symmetric keratotic papules localized in the seborrheic areas. The estimated prevalence of the disease is 1 in 55 000^1^ to 1 in 100 000.^2^ The mechanism of DD includes a loss of adhesion between adjacent epidermal cells and abnormal keratinization. Here, we describe a rare case of unilateral segmented DD associated with several neuropsychiatric disorders.

## 2 CASE REPORT

**Figure 1:**
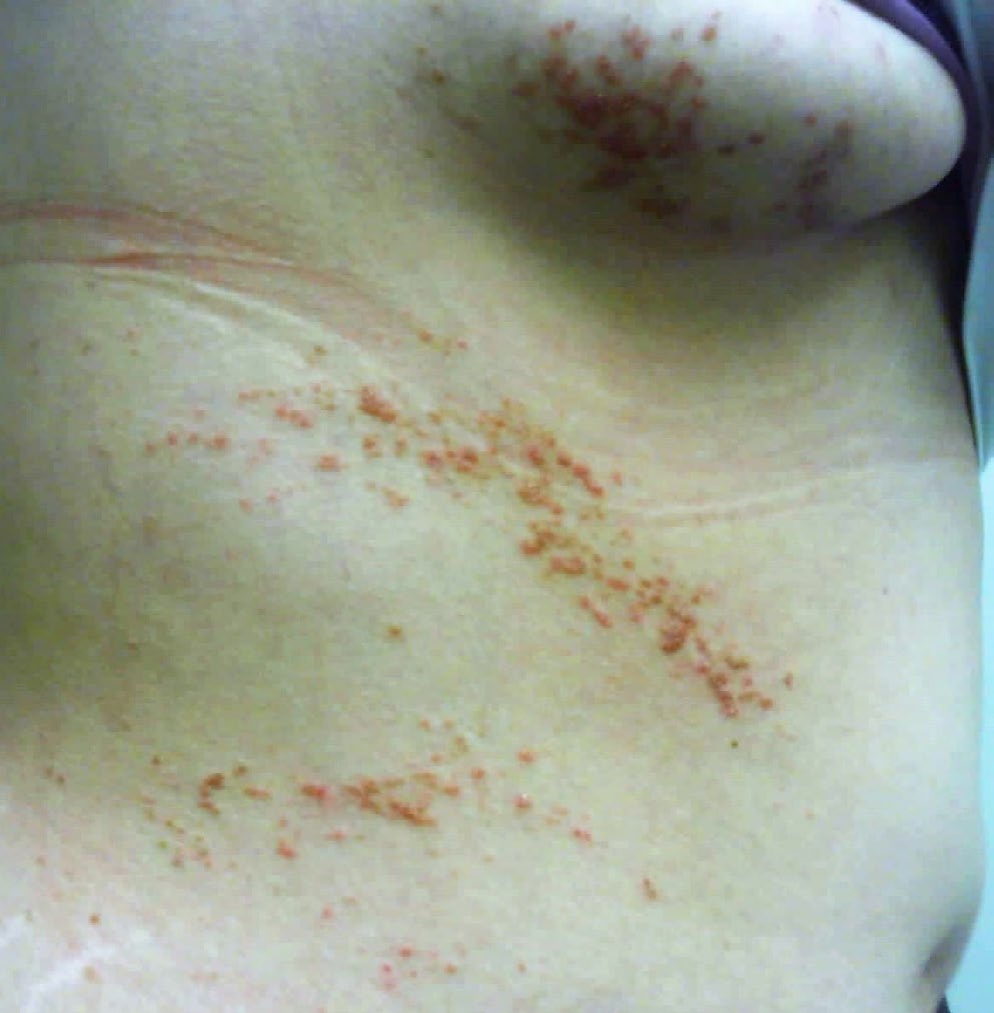
Hyperkeratotic erythematous papules confined to the right side of the body following lines of Blaschko

A woman in her thirties presented with scaly papules on the right neck and trunk for several years. The rash was initially localized to her abdomen but spread to the chest and neck. It was pruritic and refractory to over the counter moisturizers. Family history of similar lesions was negative. Physical examination revealed erythematous scaly papules on the right side of the neck, inframammary fold, abdomen, and lower back in a Blaschkoid distribution (Figure 1). The fingernails and toenails were without nicking or splitting. Past medical history was positive for multiple neuropsychiatric disorders: ADHD, anxiety, bipolar disorder, and depression.

**Figure 2:**
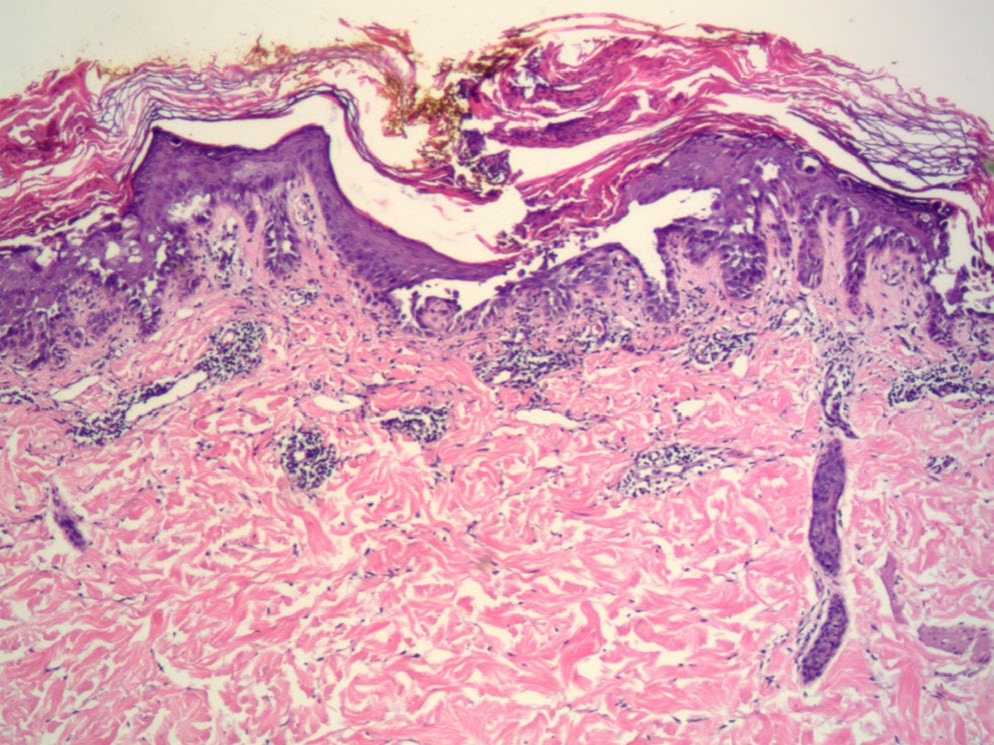
Histopathologic examination shows suprabasilar acantholysis, corps ronds and grains (hematoxylin‐eosin, original magnification × 25)

**Figure 3:**
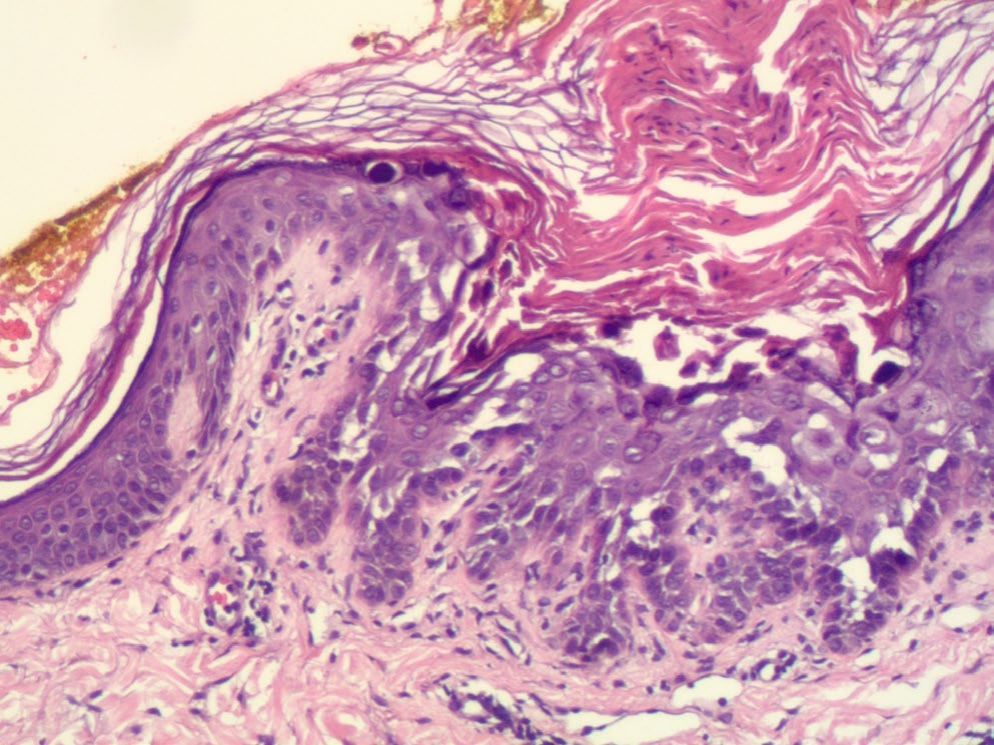
Histopathologic examination shows suprabasilar acantholysis, corps ronds and grains (hematoxylin‐eosin, original magnification × 100)

Topical steroids were prescribed with slight improvement in pruritus but the papules remained. A biopsy was obtained for histological analysis and showed foci of suprabasilar acantholysis and dyskeratosis with corps ronds and grains (Figures 2 and 3). After biopsy, a topical retinoid (Retin‐A ointment 0.06%, Valeant Pharmaceuticals, North America LLC, Bridgewater) was prescribed and the papules improved.

## 3 DISCUSSION

The genetic origin of the Darier's disease has been localized to the ATP2A2 gene on chromosome 12q23‐12q24 which is responsible for the sarcoplasmic/endoplasmic reticulum ATPase Ca2+ pump.^3^ The defective Ca2+ pump leads to deficiency of Ca2+ at the cell membrane and abnormal P‐cadherin protein expression in the desmosomes, causing impaired cell‐to‐cell adhesion that leads to the acantholysis upon histologic examination.^4^, ^5^, ^6^

It is thought that a postzygotic somatic mutation in the ATP2A2 gene leads to the genetic mosaicism and unilateral expression along Blaschko's lines characteristic of unilateral Darier's disease.^7^ Patients with localized DD develop skin lesions in the third or fourth decade of life, without a family history of the disease. They typically have no other associated features of DD, such as nail fragility causing characteristic V‐shaped nicks, palmar signs, or mucosal signs.^8^

Interestingly, Darier's disease has been correlated with neuropsychiatric disorders including epilepsy, depression, and an increased risk for developing intellectual disability.^9^, ^10^ It is thought that the link between the skin and brain tissue is due to the tissues common ectodermal origin and use of intracellular calcium stores involved in neuronal excitability and signaling that could cause neurological disturbances as seen in our patient.^9^

The differential diagnoses of an acantholytic dermatosis include Hailey‐Hailey disease, which occurs in the intertriginous areas, and Grover's disease, which occurs in the trunk but is typically bilateral and transient. DD presents with a positive family history and is found in the seborrheic areas as well as having lesions in the nails, mucosa, and acral lesions including palmar pitting.^5^ The unilateral distribution of the rash, a negative family history, and a lack of nail, mucosal, or palmar lesions indicate a diagnosis of unilateral Darier's disease.

Treatment of DD typically consists of oral and topical retinoids. This is effective due to the decreased turnover of cells in DD which is seven times slower than that of healthy cells.^5^ Our patient responded well to topical retinoid administration; thus, oral retinoids were not given due to their significant potential for toxicity. The diagnosis of unilateral DD is challenging, but should be considered in the differential diagnosis of an acantholytic dermatosis in a unilateral distribution and a history of a neuropsychiatric disorder.

## CONFLICT OF INTEREST

None declared.

## AUTHOR CONTRIBUTIONS

LH: is a main author and researcher. DM:is a co‐author and researcher.
